# A day treatment program for adults with eating disorders: staff and patient experiences in implementation

**DOI:** 10.1186/s40337-019-0252-4

**Published:** 2019-07-01

**Authors:** Kylie Matthews, Leanne Gordon, John van Beusekom, Jeanie Sheffield, the QuEDS collaboration group, Elaine Painter, Rachael Bellair, Warren Ward, Susan Patterson

**Affiliations:** 1 Royal Brisbane and Women’s Hospital, Nutrition and Dietetics, Level 2 James Mayne Building, RBWH, Herston, 4006 Australia; 2 Psychology, University of Queensland, St Lucia, 4067 Australia; 3 Queensland Eating Disorder Service, 2 Finney Road, Indooroopilly, 4068 Australia; 4 Royal Brisbane and Women’s Hopsital, Mental Health Centre, J Floor, Herston, 4006 Australia

**Keywords:** Eating disorders, Anorexia nervosa, Day treatment program, Patient experience, Service provider experience

## Abstract

**Background:**

Eating disorders are serious conditions which are increasing in prevalence internationally. The causes of these conditions are complex and incompletely understood, and clinical presentations can vary over time. The complexity of these conditions can also complicate treatment. Therefore, stepped care treatment comprising a hierarchy of interventions, including access to day treatment programs (DTPs), is recommended. While studies have examined patient outcomes and provided narrative accounts of these programs, no published studies describe DTP development. This study aims to address this gap by examining development and implementation of a DTP from service providers and patients’ perspectives.

**Methods:**

This study utilised a mixed-methods design to examine the design and implementation of a publicly funded, closed group DTP in Australia. Data from service records and documents were analysed, alongside interviews with patients and interview and focus groups with service providers conducted between June 2016 and July 2017. Quantitative data were analysed using descriptive statistics. Qualitative data were analysed using the Framework Approach.

**Results:**

Seventeen service providers (*n* = 4 in managerial and *n* = 13 clinical positions, with clinical experience of 3 months to 20 years) and 11 patients (100% F, 17-33 years) were interviewed. The service providers reported that implementation was a stressful undertaking due to tight timeframes to achieve multiple tasks. Patients had diverse opinions regarding the DTP content and the group treatment experience. Despite this, all patients reported benefits from attending the DTP, varying from improvements in mood, weight gain, development of personal skills and strengths, to living independently. For further benefit, patients suggested that programs could be shaped and targets towards differing patient groups, with fewer breaks throughout treatment.

**Conclusions:**

Designing and implementing a DTP is a challenge and can be a time-intensive undertaking, however the result can be beneficial for both service providers and patients. The closed group format was beneficial in creating a supportive environment, though may have led to increases in additional eating disordered behaviours. While the current structure of this DTP may require reconsideration, organisations considering implementing a new DTP may find usefulness in the overall design described in this study, alongside learning from the issues experienced.

## Plain English summary

This study aimed to describe service providers and patients’ experiences of the design and implementation of a new day treatment program (DTP) for those patients with eating disorders. Interviews and focus groups were conducted, alongside data drawn from service records and documents. Interviewed service providers reported that designing and implementing the DTP was challenging and time-intensive, however did exhibit pride when discussing the DTP produced. Patients reported diverse opinions regarding the content and group treatment, however all reported some benefit from participating in the DTP. While changes to the current DTP may be required, organisations considering commencing a new DTP may find the design described useful, particularly with the incorporation of the issues experienced by the service providers and patients during implementation.

## Introduction

Eating disorders (EDs), including anorexia nervosa (AN) and bulimia nervosa (BN), are serious, potentially life-threatening conditions, which are increasing in prevalence internationally [1]. The causes of these conditions are complex and incompletely understood, with aetiology in individuals likely related to unique combinations of genetic, biological, psychological and social factors [2]. The clinical presentations and experiences of people with a given diagnosis are highly individual and commonly vary over time. Co-morbidity of other psychiatric conditions (including depression, anxiety, borderline personality disorder, obsessive compulsive disorder, drug and alcohol dependence) is experienced by most (up to 97%) patients with AN and BN [3]. Moreover, EDs are commonly associated with debilitating behavioural and social problems [4].

The complexity of EDs, the typically ego-syntonic nature of symptoms, and co-morbidities complicate treatment. While some interventions used in children and adolescents receive strong empirical support, beyond specification that treatment be provided in the least restrictive environment, preferably in an outpatient setting, no extensively validated intervention has been identified for adult patients [5, 6]. Hence health professionals employ diverse interventions alone or in various combinations dependent on the symptoms and severity of the disorder and the health status, circumstances and needs of the patient [2]. Response to treatment is often inconsistent, and relapse is common (up to 52%) [7–12]. Collectively and individually people with EDs may require a spectrum of care ranging in intensity and focus. Strategic documents in Australia and other jurisdictions recommend stepped care models comprising a hierarchy of interventions ranging from advice and psychoeducation to intensive inpatient care, with individuals able to access support appropriate to their needs [3, 13, 14]. The proposed hierarchy includes day treatment programs (DTP) posited both as a means to forestall or preclude the need for admission to hospital and enabling sustainable recovery from EDs following acute treatment [3, 13, 14]. DTPs of varying intensity and duration, employing diverse packages of interventions, have been implemented in jurisdictions across the western world. The limited research available demonstrates that participation can have therapeutic benefits with reduced psychosocial impairment, positive adjustment in attitudes, body-weight restoration and decreases in binge/purge symptoms reported [15–23].

While studies have examined patient outcomes and provided narrative accounts of these programs, no published studies describe development of a DTP; an understanding of the views and experiences of the health professionals implementing programs (service providers) is lacking and little is known about the patients’ experiences of treatment in these programs. The absence of this information constrains design of DTPs, potentially leading to suboptimal practice and inefficient use of resources. This paper aims to address this gap by examining the development and implementation of a program from service providers and patients’ perspectives. The aspiration is that learning in a given context can inform development of the subject DTP and implementation of similar programs in other localities.

## Methods

### Study context

This mixed-methods study examined the design and implementation of a publicly funded DTP in Queensland, Australia. Pursuant to National Standards recommending establishment of stepped care models, the Queensland Government awarded funding to the support, establishment and operation of a DTP for people with EDs. The DTP was implemented by the Queensland Eating Disorder Service (QuEDS), a state-wide service in south east Queensland. Established in 2001, with two fulltime staff providing consultation liaison and case management services, the QuEDS has expanded into providing staff and carers’ education and coordinating patient intake as funding has become available. With the long-term aim of building a comprehensive ED service, the QuEDS has progressively developed state-wide consultation liaison services and outpatient treatment clinics to complement the inpatient treatment for adult patients offered at a publicly funded, specialist unit.

### Study design and aims

Seeking to develop knowledge that could be practically useful in developing DTPs and other services, the study team employed mixed methods underpinned by philosophical pragmatism [24]. Quantitative and qualitative data collected from service documents and records were combined with qualitative data collected from service providers and patients to describe:

(i) design and implementation of the service
(ii) service provision over 12 months
(iii) implementation from the perspectives of service providers
(iv) perceived helpfulness and experiences of the DTP from the perspectives of patients
(v) factors potentially influencing sustainability of the DTP

This study was approved by an authorised Human Research Ethics Committee (HREC/16/QRBW/273) and because the study involved students as part of the research team, the University of Queensland’s Behavioural and Social Sciences Ethics Committee (Approval: 2016001019).

### Data collection

Data were collected from service documents and records, and in semi-structured interviews and focus groups with staff involved in design and/or delivery of the program. Patients participated in individual semi-structured interviews only.

### Service data

Service records and documents were provided to the study team for review and analysis. Service documents and records from which data were collected included tender and funding documents, minutes of QuEDS meetings, and DTP treatment plan templates and timetables. Data regarding service provision were obtained from routinely kept records of program referrals, patient engagement and completion.

### Interviews and focus groups

Service providers involved in the establishment of the DTP and provision of the initial four 12-week programs and patients enrolled in one of these programs were eligible to take part in the interviews or focus groups. Recruitment and data collection were conducted in two rounds, in June–July 2016 and June–July 2017.

Service providers self-referred to participate in this study following dissemination of information through routine communication channels including weekly staff meetings. Patients were provided with written and verbal information about the study at their initial intake assessment and invited to consider participation in one or both of two ways: by giving permission for routinely collected data to be provided de-identified to the research team and/or by taking part in an interview. Those who agreed to sharing data signed a consent form to that effect. Participants could withdraw from the interview at any time without penalty.

Interviews were conducted by two psychologists, one male and one female (JVB and LG), completing Masters’ degrees in Clinical Psychology. The students were supervised by two experienced health-services researchers with extensive experience working with people diagnosed with EDs (SP and JS). Interviewers used topic guides flexibly to explore the participants’ treatment journey (“How did you find your way into the DTP?”) and experiences with, and views about the DTP (“What did you think of the program as a whole?”). Patients were invited to describe their expectations and perceived impact of the DTP with reference to various treatment components. Service providers were asked about their professional experience, roles within the DTP, impact, philosophy, rationale and evidence base of the DTP, and development and delivery of the program. Questions of the service providers included, “What were the biggest challenges in developing the DTP?” and, “What is your understanding of the patients journey throughout the DTP?” All patients and service providers were asked, “Would you refer a friend to the DTP?” and were invited to identify potential improvements for the DTP at conclusion of the interviews or focus groups. All interviews were audio recorded with permission and transcribed verbatim for analysis. Patient interviews lasted between 45 and 60 min. Service provider interviews and focus groups lasted between 30 and 60 min.

### Data analysis

Quantitative data from service records were entered onto SPSS [25] for descriptive analysis.

Qualitative data from interviews were analysed in three phases with findings of separate analyses of data from each round of interviews, subsequently synthesised with reference to original data. Led by KM, the synthesis employed the Framework Approach [26], selected as it enables development of responses to a priori research questions while remaining open to concerns of participants not anticipated by researchers. Both deductive and inductive logic were employed to reduce and synthesise original data and results of initial analyses. First, familiarisation was achieved by repeated reading of transcripts of all interviews and the Masters Theses reporting the initial analyses. Data were then coded descriptively before being allocated to an initial frame, in which cells corresponded to source by research question (with multiple allocations permitted) or ‘other’ (data considered not relevant to question). A process of constant comparison was then used to iteratively develop case- and question-based summaries of data. Patterns and exceptions were identified with similar data grouped and descriptively labelled to produce sub-categories. In this manner, analysis developed contextualised collective responses to research questions as narratively presented below. Rigour in analysis was promoted by checking developing findings in original transcripts and ongoing dialogue among authors who drew on differing disciplinary and research expertise to ensure the account remained grounded in data.

## Results

Results are structured into two sections. The first examines the implementation of the DTP, integrating data from the service provider interviews and document analysis. As described below, service providers, challenged to plan a program in the absence of a best practice model and within funding constraints found design implementation a stressful experience. The second section examines the patient experience of the DTP. Patients found different aspects of the DTP helpful and reported varying experiences of the structure and content of the program. However, the majority of patients interviewed had, or would, recommend the DTP to a friend.

### DTP funding, design and structure

Data were collected in interviews with 17 service providers (71% of 24 eligible staff) in two focus groups and five individual interviews). Six service providers chose not to participate and one was unavailable at the time of data collection. The majority of service providers were female and held various managerial (*n* = 4) and clinical positions (*n* = 13). Service providers had varied disciplinary backgrounds (nursing, allied health, and medical) and experience working with patients with EDs of between three months and 20 years. Several reported active participation in national associations and interest groups concerned with treatment of EDs.

### Funding and design of the DTP

Funding was awarded for establishment of the DTP in July 2015, conditional on launching the program within 12 months. As described by those involved, this necessitated expedient achievement of multiple interlinked activities, including securing a venue, recruiting staff, and critically, designing the structure and content of the DTP. While tasks were allocated to various team members, interview data indicate an ‘all hands on deck’ approach, dispersed leadership and extensive consultation within the team and with various stakeholders. Multiple activities were undertaken concurrently.

Securing an appropriate venue within the allocated budget proved a key challenge, necessitating reallocation of a substantial part of the staffing budget to support development of the best available option. As noted by one manager,

*“…we applied and received the money before we had a facility to run it in. So, then we had to find a suitable space, which was very difficult…probably a mistake...”* Service provider 1

A senior psychologist was tasked with researching and formulating a therapeutic program to achieve program goals articulated in funding documents: providing patients with supported nutrition and skills to enhance their psychological recovery and enable appropriate follow up. While consensus among service providers was that a group format and supportive meal therapy were ‘givens’ from the outset, their accounts demonstrate a protean approach to program design, with ongoing refinement reported over the evaluation period. Service providers reported that initial structure and clinical interventions were influenced by knowledge of ‘what was done in other settings’, skills and preferences for various therapeutic modalities of staff involved, and evidence.

“…*a lot of the content has come from evidence based individual therapies…and then we’re doing this adaptation to a group format…Wise choices is an established group program…but it’s not an eating disorders based treatment…so one of our groups has evidence base for groups, but not eating disorders.”* Service provider 2

Acknowledging the partial and inconsistent nature of evidence in treatment of EDs, service providers conceptualised the program as “*evidence building.”*

To assess impact of participation on patients, service providers reported that a range of assessment tools were selected. Tool selection was informed by ‘common practice’ in treatment of EDs (e.g., Eating Disorder Examination Questionnaire), and by the various professions involved. For example, a key concern was enabling comparison of outcomes with other therapies provided by the QuEDS (e.g., Cognitive Behavioural Therapy – enhanced [CBT-e]).

Interview data suggest some uncertainty among service providers regarding the process and rationale for determining patient eligibility criteria and subsequently, application of those criteria over the course of the evaluation. All involved in DTP design agreed that (in addition to diagnosis of an ED), enrolment was conditional on medical stability, with criteria to assess this defined by the Royal Australian and New Zealand College of Psychiatrists [27], and Body Mass Index (BMI) (≥16 kg/m^2^ for patients aged ≥18 years or appropriate BMI percentile range < 18 years old). Patients could be referred to the DTP by their General Practitioner (GP) or mental health treating team. Enrolment was contingent on commitment to weekly consults with GPs for the duration of the program. While ‘stage of change’ was not specifically included in the eligibility criteria, service providers described incorporating informal assessment of ‘readiness’ or potential to benefit from the DTP. From the outset, underweight patients were encouraged to achieve weight restoration during treatment.

Service providers shared the view that design and implementation of the DTP was a difficult and stressful undertaking. It was also acknowledged that colleagues at other sites in Australia had advised that the initial 12 months would be demanding and time-consuming. Service providers described feelings of unpreparedness for the size of the task despite these discussions.

*“…we went through a period of about five months where we felt sick with anxiety…so many uncertainties, and the premises we were moving to aren’t quite big enough, and, I think there was a period where a lot of us were duplicating or triplicating things, and we didn’t have a very good structure or coordination.”* Service provider 3

### The structure of the DTP

As outlined in Table 1, the DTP was a closed group program, structured into three separate phases. The closed group format was chosen by the service providers due to their knowledge of group curative processes, including the development of socializing techniques and interpersonal learning. As the DTP was designed as a sequential therapeutic program, it also would have been difficult for participants to commence the DTP after the initial week. The DTP was designed to run four times per year with a maximum of eight patients per program. The service providers reported that eight participants was considered small enough to enable group cohesion but large enough to justify resources.

**Table 1:** Description of the phases incorporated into an DTP commenced in Brisbane, Australia

DTP Phases	Description of phases
Phase one (weeks 1–2): Assessment and treatment planning	Incorporates five individual assessment sessions with a dietitian (2), key worker (2) and psychiatrist (1). Participants work with program staff and designated Key Worker to develop an Individualised Treatment Plan (ITP). The ITP establishes treatment expectations, treatment non-negotiables (for example, the expectation to eat with other group members and staff) and personal goals of participation. Throughout the program, participants meet with their Key Worker to assess progress and refine the ITP.
Phase two (weeks 3–10) (including one week break in week 5): Supportive Meal Therapy and group program	Supportive meal therapy and meal challenges in conjunction with various therapeutic interventions: Maudsley Anorexia Nervosa Treatment for Adults (MANTRA), Cognitive Behavioural Therapy – Enhanced (CBT-e), Cognitive Remediation Therapy (CRT), Acceptance and Commitment Therapy (ACT), Wellness Recovery and Action Plan (WRAP), peer mentoring, diversion/distraction/self-soothing/relaxation strategies, music therapy, therapy dog visits, and recreational groups. Group sessions are facilitated by clinicians.
Phase three (weeks 11–12): Discharge planning	Participants discuss their ongoing treatment options with program staff. Five individual appointments are conducted with a dietitian (2), key worker (2) and psychiatrist (1).

When discussing the program structure, service providers recognised the impact of participation in an extended treatment program on patient’s usual activities, noting that lives would in many ways be ‘put on hold’ while attending the DTP, with decreased or no attendance at university, employment, and social outings. This knowledge influenced decisions regarding the length of the program, with 12 weeks deemed enough time for education and treatment while also being an appropriate timeframe for those patients studying at university to attend during summer holidays. Service providers provided justification for the week off in the middle of phase two:

*“One was to give people a bit of a chance to practise some of the skills they learnt. So, it was a patient benefit and a staff benefit...We were just finding they were getting so tired and it’s a very intensive challenging program. Just needing a bit of a rest really in the middle, for everyone.”* Service provider 2

The length of the DTP was also influenced by requests of the service providers. All service providers reported concerns regarding prolonged contact with patients during multiple, recurrent sessions of supportive meal therapy. Further to this, different attendance times for each day of treatment were chosen to allow for ease of supportive meal therapy at different mealtimes (for example, Monday 11 am to 7 pm and Friday 7.15 am to 2.30 pm). Patients were expected to attend the DTP on Monday, Tuesday, Thursday and Friday during Phase two (see Table 1), for approximately 7–9 h per day. Time spent in different therapeutic interventions, free time, appointments with key workers and in meals would differ each day. As an example, on Mondays patients spend 30 min listening to a TED talk prior to 30 min for lunch. This is then followed by 90 min with peer mentors before progressing with free time, afternoon tea, diversion therapy, cooking, dinner and reflection time.

Patients were requested to provide their own food, with the exception of meal challenges held at restaurants and cafes. All food that the patients supplied were checked for nutritional adequacy.

### Staffing

Implementing the DTP required recruitment of four additional staff members and reallocation of duties to some already employed within the QuEDS. Service providers agreed that a multidisciplinary team was essential in providing an effective program, but in the absence of a best practice workforce model and with limited funding, staff were recruited based on what managers considered would make a cohesive team. Approximately eight staff members were required to run the DTP, however their time was also split across various other outpatient clinics.

The ‘stress’ involved with implementation and its impact on job satisfaction and personal wellbeing were thought by stakeholders to have contributed to high staff turnover during the first 12 months of program delivery. Adding to this, during the initial DTP there was heavy reliance on the shopping centre across the street for both amenities and last minute purchases for daily activities.

*“It* [burnout] *was multifactorial. There was a lot of upheaval in the environment and renovations. We didn’t have use of toilets for a lot of it.”* Service provider 2

*“You’re there early in the morning revising what you’re doing in your group for the day. And realising ‘oh we don’t have this’ and running over to the shops…”* Service provider 2

### The delivered DTP

Four programs were delivered between May 2016 and May 2017. All 27 patient referrals received during this time were accepted for treatment. The program was completed by 20 with seven discontinuing participation in the initial three weeks (*n* = 4) or later in the second phase (*n* = 3). Reasons given for discontinuation included anxieties around treatment (*n* = 2), medical instability (*n* = 1), opting for alternate treatment (*n* = 1), an unplanned holiday (*n* = 1), and a worsening mental health condition (*n* = 1).

### Developments during evaluation

While maintaining that the philosophy and core components of the DTP remained, service providers reported various refinements of the program over the course of the evaluation. Service providers reported that integrating consumer feedback became an important influence in adapting components of the DTP. An example of this was the addition of a carers’ support program from the third DTP onwards after an expressed need for further support from family members and carers.

While patients were encouraged to achieve weight targets during the initial three DTPs, low achievement rates led to these weight targets becoming a non-negotiable condition of participating in DTP treatment for the fourth program. The service providers reported that the creation of this non-negotiable condition also assisted in ensuring patients recruited into the DTP were at an appropriate stage of change for treatment.

Other adaptations included removing some social activities, such as bowling, that were incorporated into the initial DTP. Scheduling changes were also made due to patients finding some days ‘too intense’. Finally, the frequency of staff assisting with supportive meal therapy was reduced due to staff requests. Overall, service providers felt that the initial programs experienced difficulties and were implemented with a limited number of policies and procedures.

The majority of service providers reported that they would recommend the program to a friend, with only one suggesting that treatment needs to be matched to patient needs:

*“…I would only do that with very big caveats about ‘do you really want to do it, it’s very difficult, you might have some unexpected consequences and triggers and things’. I would have to seriously think about it if it was a friend of mine.”* Service provider 2

### The patient experience

Eleven patients completed interviews. All participants were female, aged 17–33 years, with most under 22 years. Nine reported a diagnosis of AN, with either binge-purge or restrictive characteristics; two patients chose not to disclose their diagnosis. With duration of illness between three and 19 years, all described multiple treatment episodes including a range of outpatient psychotherapies; all but one had been hospitalised for treatment for their ED. Seven patients reported commencing the DTP within a month of discharge from inpatient treatment, one attended the DTP as an alternative to recommended inpatient treatment, and three attended to trial a different treatment option. Patients reported varying expectations and anticipated outcomes from participating in the DTP. While some expected the program to enable full recovery, others were less ambitious, anticipating progress in recovery journeys. Despite varying expectations, most patients reported experiencing the DTP as helpful. Patients’ spoke of developing skills and feeling better equipped to manage their EDs. The majority continued CBT-e treatment after the DTP concluded.

*“I did go into the program expecting to be cured and obviously haven’t come out cured. It’s helped in terms of its kept me going so far and given me some of the skills emotionally and psychologically I think that’s something that takes more time…But I think it was really useful”* Patient 9

*“I knew that I wasn’t going to come out recovered, I just wanted more tools in my toolkit.”* Patient 2

Patients commonly reported experiencing wide ranging emotions over the course of program participation. They spoke individually of feeling supported, frustrated, anxious, happy, challenged, motivated, and misunderstood. Most reported anticipatory anxiety, linked to uncertainty about what was ahead, the prospect of prolonged contact with the other patients and of gaining weight.

*“I was so scared…I’m going to get so fat…it’s going to be torture, they’re going to make me eat excessive amounts of food, and it’s going to be hell, and…I’m going to be the biggest one there...”* Patient 1

### Supportive meal therapy and food-related components – experiences and views

Patients reported finding supportive meal therapy beneficial, and considered it a key step in their recovery journeys. Benefits discussed included structuring of meals throughout the day and normalising eating socially. However, patients did report difficulties with other food-related parts of the program, for example, experiencing an increase in ED cognitions during cooking classes. Providing their own meals according to specifications set by the dietitian also proved challenging at times.

*“I found it quite confronting to have my meals checked. Like, I’ve never really had anyone check my meals and mark you off and give you extra if you haven’t bought the right thing. I just found that very, yeah quite confronting that it was done in front of everybody.”* Patient 8

Patients treated in the initial DTP program reported that restaurant meal challenges were difficult, however patients in the following programs reported that they desired to be pushed harder during meal challenges despite no changes being made to this component of the program.

### DTP content

Asked about therapeutic content, patients expressed divergent views, with experience of EDs and treatment influencing perceptions. For example, some expressed that the content was too simple and not appropriately targeted:

*“…you know with the older, longer experience of eating disorders, there is more of a shift of focusing on quality of life, rather than eating disorder symptoms or dietary information...”* Patient 6

More commonly, patients found the content informative and beneficial in creating behavioural changes. Patients who did report positive experiences with the content also acknowledged that perceived intensity of some sessions made engagement difficult.

Patients volunteered commentary on different therapies and the peer mentoring incorporated into the DTP. Divergent views were expressed regarding Cognitive Remediation Therapy (CRT), Maudsley Anorexia Nervosa Treatment for Adults (MANTRA) and Acceptance and Commitment Therapy. With respect to CRT, four patients reported needing additional guidance to understand the relevance of the activities included, whereas three patients had voluntarily continued some of the activities and board games incorporated into the CRT sessions after completing the DTP. Five patients reported finding MANTRA confronting, particularly in the group setting, but useful in terms of examining the effect of EDs on personal relationships. Patients commonly reported that discussions examining mindfulness and life values, the Wise Choices program, and sessions with peer mentors were useful.

[with respect to peer mentors] *“I think it was really good to kind of have someone who knew where you were coming from…all the health professionals were very good in their roles but to have somebody there who had just been there and got through it could kind of give you like little tips or strategies for something that you were really struggling with…that was like a key part.”* Patient 8

### Duration and structure

With a single exception, patients described the program as too short, which impacted patients’ perceptions of helpfulness.

*“…it was long enough to learn the tools but it wasn’t long enough to put them into practice…”* Patient 2

Patients shared the opinion that having discontinuity in the program, including both the day off in the middle of the week and the week long break in phase two, was unhelpful. Time away was commonly described as disruptive of process and negatively influencing progress and engagement.

*“…not compensating when you went home was probably the hardest thing…The challenges themselves were hard, but like, not compensating for them afterwards was even harder*.*”* Patient 2

*“…at one point I didn’t want to particularly come back because I had gone back to more sort of eating disorder behaviours in that break, so I was less motivated in the last four weeks…”* Patient 9

### Group dynamics and the DTP environment

Patients reported differing views regarding group dynamics. Most experienced the group as generally supportive and feeling connected to other patients.

*“…They definitely made me feel less alone in the whole process…I think that was a big thing…out of the whole program that’s what I really got out of it. Like a sense of connectedness with…people who were experiencing the same thing.”* Patient 8

However, five patients spoke of competitiveness among participants affecting group cohesion and personal recovery. Patients also spoke of cohesion and sense of belonging being hindered by perceived differences in patients’ levels of motivation, in the duration of illness and phase of recovery, in ED symptoms, and interpersonal compatibility.

*“Some people wanted to be role models, others just wanted to be the sickest, so it was kind of a challenge.”* Patient 1

A minority spoke of increasing ED related behaviours as a result of ‘learning’ from other members and a sense of competitiveness.

*“I think it made it sort of, easy for me to maybe take on other* [eating disordered] *behaviours, if I felt like that was what was expected of me…”* Patient 5

*“…trying to eat the least as possible and all that sort of stuff…trying to bring the smallest amount of food.”* Patient 11

Despite group issues, patients still found the environment beneficial. While renovations negatively impacted the service providers’ experiences of the initial DTP, with no access to on-site bathrooms or gardens, interviewed patients were still unanimous in reporting that they enjoyed the setting in all four DTPs.

*“…I loved coming here and sitting with the cushions and the garden outside…we planted those things and we would go out there and water them.”* Patient 7

### Location and access

Patients typically found the DTP easy to attend via public transport. While patients did acknowledge that participation in the DTP disrupted and intruded into other aspects of their lives (primarily university studies and time with their social group), they also acknowledged that prioritising health was a necessity. The DTP was commonly reported as being worth the time it took to complete.

*“…in a way it was permission to look after myself when I was here. Whereas when I was at home I’d do it but I’d just feel so bad because I don’t have, I don’t feel like I have the support to do it, and I don’t feel like I deserve to do it because no one is here telling me I should…”* Patient 5

Patients also expressed gratefulness for the publicly funded access to treatment.

*“…it’s good that it’s funded by the government too because a lot of people with eating disorders are so sick that they can’t work so they don’t have the money to afford private treatment...”* Patient 10

### Life after the DTP

Notwithstanding varied experiences with the DTP content and group dynamics, patients were unanimous in reporting that all had gained a positive result from attending the DTP. Reported benefits included improvements in mood, weight gain, and development of personal skills and strengths, with one reporting program participation had enabled her to live independently for the first time in ‘years.’ The program was compared favourably to outpatient treatments.

*“I’ve come such a long way in such a short amount of time compared to the weekly appointments I was having with my dietitian and my psychologist.”* Patient 8

However, most patients reported difficulty adjusting when the program concluded, with a loss of routine and structured plans for their ongoing treatment. Patients also reported difficulties in managing their behaviours and emotions with a decrease from four days of contact per week to less frequent outpatient therapy.

*“…I struggled a bit for the first couple of weeks after it ended…because there was so much uncertainty in what was going to happen next with my treatment…”* Patient 5

*“…it was kind of terrifying. We had the four days a week and it kind of went to nothing, so that was a little hard.”* Patient 4

*“…you leave DTP and…you’ve got the whole day, while you adjust back to life to be with the thoughts, here* [the DTP venue] *you have distractions, so I feel like they* [ED cognitions] *were actually around more.”* Patient 3

The majority of patients said they would, or already had, recommend the program to a friend. However, one expressed reservations about ‘fit’ and relevance of the program for people with extensive experience of EDs.

*“…thinking of some of my friends who might be…in their forties, no. Some of them might really need it but I can’t imagine what it would be like to…have a longer experience with an eating disorder and sitting here and hearing this stuff from staff members who have such a small experience of eating disorders compared to you...* ”Patient 6

### Ongoing requirements

All service providers agreed that the DTP should continue however also identified three ongoing needs, including additional referrals, continued and potentially increased funding to enable recruitment and retention of appropriately experienced staff, and the potential need for accommodation for patients. This final need was acknowledged as while the DTP was created to provide treatment to individuals across the state, there are difficulties in servicing areas outside of the immediate vicinity of the DTP.

*“…we’re a state-wide service, but I guess the reality is that it’s not available…there was pressure from within those services for us to take people, but we don’t have any accommodation attached, so even though it’s a state-wide service, logistically it’s really a service for the South East and probably more so Brisbane…it does create a bit of inequity in terms of service access…”* Service provider 5

Patients suggested that programs could usefully be shaped for or targeted to different patient groups dependent on motivation, experience with EDs and life circumstances.

## Discussion

To the authors’ knowledge, this is the first study to explore design and implementation of a DTP for people diagnosed with EDs, from the perspectives of both staff and patients. This study found that, with tight timeframes to achieve multiple tasks, service providers experienced implementation as a difficult and stressful undertaking. They were however proud of the program and its delivery. Patients in all four programs found the DTP beneficial in different ways to their recovery journeys, despite differing viewpoints on the content delivered.

### Knowledge gained from the implementation of the DTP

Despite DTPs being integral components of treatment for EDs in North America, the United Kingdom and in other states in Australia, the evidence to support the use of day therapy treatment for individuals with EDs remains scarce [15]. Similar to other day programs described in the literature, this DTP has incorporated core evidence-based individual psychotherapy treatments and integrated these into a group format alongside supportive therapies using clinicians’ experience and knowledge [15]. While the patients variously perceived therapies as helpful, the only therapy that was unanimously identified as beneficial was supportive meal therapy. These findings indicate that certain therapies might need to be matched to certain patient characteristics and/or that further research is necessary to assess the benefits of the evidence-based psychotherapies adapted to group format for the DTP. There is also a need for further research of the therapies utilised that currently have no evidence for use with patients with EDs. However, due to the highly variable and complex nature of EDs, alongside considerations of other treatments and medications that patients may be taking, it is acknowledged that evaluating these therapies with this population group will be an ongoing challenge.

Willinge, Touyz and Thornton [23] identified another challenge for day programs: the consideration of outcome criteria directly related to treatment goals as a means of evaluating whether programs are achieving what they intend to. This limitation is replicated in this program; in the initial 12 months of the DTP, treatment goals were loosely defined and evaluation methods were not targeted towards these goals. Further consideration is required by the service providers and those commencing new DTPs to establish clear objectives so that appropriate outcome measures can be identified and evaluation can be performed.

Service providers found designing and implementing the DTP a stressful experience. This may have led to high staff turnover, which can become a problem with relatively few clinicians who specialise in the treatment of EDs. These experiences should be acknowledged by individuals considering a similar undertaking in the future including policy makers, funders, and practitioners. The DTP design and structure may have been able to be addressed prior to funding. However, due to the unpredictable nature of obtaining funding for new ventures, there may be difficulties in achieving this and in alleviating the pressure staff experience during the implementation of new programs.

### Considerations for moving forward

Internationally, day programs have treated patients for periods of time anywhere between three and 39 weeks, with Abbate-Daga et al. [15] suggesting that this implies there are two different levels of treatment. Whereas shorter programs are symptom-focused, longer duration day programs focus on patients’ relational skills, psychodynamic symptom understanding and more gradual changes in body weight [15]. All but one patient in this study identified that they desired a longer program. While there is limited evidence that programs of any length are universally helpful, it may be prudent to consider either extending the current DTP or designing a second longer program, particularly for those individuals with more severe or longstanding forms of EDs, or for those who self-identify as believing they may benefit from further assistance. Previously and in support of this, Thornton, Beumont and Touyz [28] identified that there is a need to offer a complete continuum of outpatient care starting at five days per week and gradually tapering to one day. Additional support for five days per week is reported by Olmsted [29]: while weight gain was not significantly different between participants attending a day program for four days per week and those attending five days, participants did demonstrate fewer disordered eating behaviours when attending the more intensive program. Incorporating these recommendations may address the patients’ requests for increased contact with service providers following the four days of continuous contact for eight weeks, and the reports of increased compensation on days away from treatment. However it may result in delays in treatment for the next intake of patients. As an alternative, the role of aftercare or the transition process between intensive DTP and less supportive outpatient services may require further investigation [23].

This service did not assess patients’ capacity to relate in a group setting. In the future this may be beneficial in accounting for group relational dynamics [15]. Despite applying eligibility criteria, there was also a dropout rate of 26% in the initial four programs. The authors speculate that these dropout rates may indicate that patients require additional support within the initial three weeks of treatment to combat issues arising during their assessment and commencement of the DTP, or that the eligibility criteria require reconsideration. As most patients identified concerns regarding what to expect from the DTP, clear outlines may be warranted for those patients experiencing anxiety with the initial process. However, high non-completion rates in other programs have been partially explained by service pressures to accept individuals who were not motivationally ready [20], and service providers in the current study did report a similar issue with lower-than-expected referrals. Despite the intensity of the DTP, dropout rates were still lower than those consistently reported for CBT-e [30], suggesting that group processes assisted in keeping patients in treatment [31].

This study includes several limitations. The authors were reliant on self-report for the majority of data collected and as with any human accounts of activities and subjective experiences, these reports are constructed within circumstances to which we are not privy. As with any analysis of qualitative data, our representation of participants’ views and experiences is vulnerable to bias. However, attempts were made to confirm representation of the service providers’ views and interpretation of these. Finally, as experiences were examined after only four programs, there is limited insight into ongoing challenges and changes in the design of the DTP, staffing, and the integration of consumer feedback which constrain transferability. However, this adds to the literature and speaks to the need to instigate rigorous and responsive evaluation processes for the ongoing assessment of the development and delivery of DTPs.

## Conclusions

Overall, this study has shown that while the design and implementation of a day program is a challenging and time-intensive undertaking, the end result can be beneficial for both patients and service providers. Unlike other day programs, this DTP found that a closed group format was beneficial in creating a supportive atmosphere, though it is unclear if the closed format led to increased adoption of additional ED behaviours. Although the treatment length and breaks currently incorporated into the DTP may require further consideration, organisations considering implementing a new day program may find usefulness in the overall design described in this study, and learnings from issues experienced by the QuEDS during their DTP implementation. However, further research should be conducted to evaluate the effectiveness of specific group therapeutic components in the context of the full range of care options to ensure patients with EDs are provided therapies that are effective in recovery.

## Data Availability

All data generated or analysed during this study are included in this published article.

## Abbreviations

ACT: Acceptance and Commitment Therapy
AN: Anorexia nervosa
BMI: Body mass index
BN: Bulimia nervosa
CBT-e: cognitive behavioural therapy – enhanced
CRT: Cognitive remediation therapy
DTP: Day treatment program
ED: Eating disorder
MANTRA: Maudsley Anorexia Nervosa Treatment for Adults
QuEDS: Queensland Eating Disorder Service
WRAP: Wellness Recovery and Action Plan
